# Bim expression modulates the pro-inflammatory phenotype of retinal astroglial cells

**DOI:** 10.1371/journal.pone.0232779

**Published:** 2020-05-04

**Authors:** Juliana Falero-Perez, Nader Sheibani, Christine M. Sorenson

**Affiliations:** 1 Departments of Ophthalmology and Visual Sciences, University of Wisconsin School of Medicine and Public Health, Madison, WI, United States of America; 2 McPherson Eye Research Institute, University of Wisconsin School of Medicine and Public Health, Madison, WI, United States of America; 3 Department of Cell and Regenerative Biology, University of Wisconsin School of Medicine and Public Health, Madison, WI, United States of America; 4 Department of Biomedical Engineering, University of Wisconsin, Madison, WI, United States of America; 5 Department of Pediatrics, University of Wisconsin School of Medicine and Public Health, Madison, WI, United States of America; Children's Hospital Boston, UNITED STATES

## Abstract

Apoptosis of neurovascular cells, including astroglial cells, contributes to the pathogenesis of diseases in which neurovascular disruption plays a central role. Bim is a pro-apoptotic protein that modulates not only apoptosis but also various cellular functions such as migration and extracellular matrix protein expression. Astroglial cells act as an intermediary between neural and vascular cells facilitating retinal vascular development and remodeling while maintaining normal vascular function and neuronal integrity. We previously showed that Bim deficient (Bim -/-) mice were protected from hyperoxia mediated vessel obliteration and ischemia-mediated retinal neovascularization. However, the underlying mechanisms and more specifically the role Bim expression in astroglial cells play remains elusive. Here, using retinal astroglial cells prepared from wild-type and Bim -/- mice, we determined the impact of Bim expression in retinal astroglial cell function. We showed that astroglial cells lacking Bim expression demonstrate increased VEGF expression and altered matricellular protein production including increased expression of thrombospondin-2 (TSP2), osteopontin and SPARC. Bim deficient astroglial cells also exhibited altered proliferation, migration, adhesion to various extracellular matrix proteins and increased expression of inflammatory mediators. Thus, our data emphasizes the importance of Bim expression in retinal astroglia cell autonomous regulatory mechanisms, which could influence neurovascular function.

## Introduction

Formation of the retinal vasculature in the mouse occurs via a finely orchestrated migration of retinal vascular cells including astroglial cells, endothelial cells and pericytes from near the optic nerve head. This is later fine-tuned with specific cell-cell interactions and remodeling. A superficial layer of retinal vessels begins near the optic disc and spreads radially toward the peripheral portion of the retina following a network laid down by astrocytic processes (first week of life) [1, 2]. Astrocytes contribute to normal retinal vascularization by mediating directional endothelial cell and pericyte migration thus establishing vascular patterning [3] and restricting the vasculature from invading the vitreous through specific signaling mechanisms [4]. Extracellular matrix proteins such as thrombospondin-1 (TSP1) can also contribute to these processes and restrict the vasculature from entering the vitreous [5]. Perturbation of these signaling events can impair retinal vascular development as occurs by disruption of VEGF signaling pathways [6]. During the next two weeks, these vessels sprout deep into the retina and spread perpendicularly to the superficial layer forming the deep and intermediate retinal vessels. By the third week of life, the retina is completely vascularized, but vascular remodeling and pruning continues for the next three weeks [1, 5].

Astroglial cells play an essential role in retinal vascular function, and provide physical support and nutrients for neurons in the central nervous system (CNS). Their foot processes envelop retinal endothelial cells in blood vessels to maintain the blood-retina-barrier (BRB) [7, 8]. The secretion of pro- and anti-angiogenic factors maintain the integrity of the CNS neurovascular function [9, 10]. Astrocytes are active participants in complex neuronal‐glial communication, synaptic signaling and regulation of blood flow, which in the CNS of adults can affect neural precursors/stem cells [11, 12]. The importance of retinal astroglial cells in maintaining retinal function is exemplified by their dysfunction contributing to various neurovascular pathologies including diabetic retinopathy a disorder that affects BRB integrity. Unfortunately, whether inappropriate modulation of retinal astroglial cell apoptosis influences these processes is not completely understood.

Modulation of survival is key not only during development but also for tissue homeostasis. Dysregulated cell survival through increased apoptosis or proliferation plays causative roles in many disease states. One manner in which dysregulated apoptosis occurs is through aberrant expression of Bcl-2 family members. Bcl-2 was the first identified member of a family of proteins shown to regulate apoptosis [13–15]. Each family member contains up to four conserved Bcl-2 homology (BH) domains through which various family members can form homo- or heterodimers to modulate apoptosis. The pro-apoptotic member Bim contains only one BH domain, BH3. Our laboratory has found Bim to be a central player modulating apoptosis of retinal endothelial cells and pericytes [16]. However, its role in modulating retinal astroglial cell apoptosis requires further delineation.

Bim expression influences cell adhesion and migration and in some cases extracellular matrix production [17–19]. We previously demonstrated that retinal endothelial cells lacking Bim expression are more adhesive and resistant to apoptotic stimuli while retinal endothelial cells lacking Bcl-2 are less adhesive and prone to apoptosis [18, 20]. Lack of Bim or Bcl-2 resulted in cell type specific opposing changes [17–21]. Even though it has been shown that apoptosis of optic nerve head astrocytes via the AKT/Bim/Bax signaling pathway leads to their dysfunction [22], little information is available regarding the cell autonomous role Bim expression plays in astroglial cells. Thus, gaining a better understanding of the role Bim plays in modulating astroglial cell adhesive and migratory function will yield important information regarding the role these cells play in retinal neurovasculature development and function.

Here we address the role Bim expression plays in retinal astroglial cell function. We demonstrated that Bim deficient retinal astroglial cells have increased vascular endothelial growth factor (VEGF) expression, decreased Akt activation, and altered matricellular protein expression. These cells also exhibited alterations in their proliferation, migration, adhesion to various extracellular matrix proteins and inflammatory mediator expression. Thus, understanding how modulating astroglial cell apoptosis influences not only normal development but also pathological ocular conditions could be therapeutically beneficial.

## Materials and methods

### Experimental animals and cell culture

Mice used for astroglial cell isolation were maintained and treated in accordance with the Association for Research in Vision and Ophthalmology Statement for the Use of Animals in Ophthalmic and Vision Research and approved by the Institutional Animal Care and Use Committee at the University of Wisconsin School of Medicine and Public Health. Retinal astroglial cells were isolated from wild-type or Bim -/- immortomice generated as we described previously [18, 19]. Mouse retinas from 4-week-old mice (6 mice) were harvested using a dissecting microscope and subsequently rinsed with serum-free Dulbecco’s Modified Eagle’s Medium (DMEM) [23]. Next, the retinas were pooled, minced and digested with collagenase Type 1 (1 mg/ml; Worthington, Lakewood, NJ) in serum-free DMEM. The cells were then rinsed in DMEM with 10% fetal bovine serum (FBS) followed by a centrifugation step at 400xg for 5 min. The cells were then rinsed in medium containing 10% serum after which they were filtered through sterile 40 μm nylon mesh (double layer; Sefar America Inc., Fisher Scientific, Hanover Park, IL). The cells were then pelleted and washed twice with DMEM containing 10% FBS. The resulting cell pellet was resuspended in 1 ml of DMEM with 10% FBS and incubated with rat-anti-mouse CD31 (Mec13.3; BD Biosciences). Endothelial cells from this preparation were pulled down by magnetic following incubation with sheep anti-rat coated magnetic beads while gently rocking for 1 h at 4°C[24] and discarded. Astroglial cells not bound to magnetic beads, were collected, washed and plated in a single well of a 24-well plate coated with fibronectin (2 μg/ml in serum-free DMEM) in growth medium (DMEM containing 10% FBS, 2mM L-glutamine, 2mM sodium pyruvate, 20 mM HEPES, 1% nonessential amino acids, 100 μg/ml streptomycin, 100U/ml penicillin, fresh heparin 55 U/ml (Sigma, St. Louis, MO), endothelial growth supplement 100 μg/ml (Sigma) and murine recombinant interferon-γ (44 U/ml; R&D, Minneapolis, MN) and maintained at 33°C with 5% CO_2_. Astroglial cells were gradually passed to larger gelatin-coated plates (1% gelatin (Sigma #G1890) in PBS) to a maximum 60 mm dish. For all experiments, astrocytes were standardly used at 70–80% confluence unless otherwise noted.

### FACS analysis

Flow cytometry was performed as we previously described [18, 23] by rinsing dishes with phosphate buffered saline (PBS) containing 0.04% EDTA followed by an incubation with 1.5 ml of cell dissociation solution (Tris-buffered saline (TBS) (20 mM Tris-HCl and 150mM NaCl; pH 7.6) TBS containing 2 mM EDTA and 0.05% BSA) to remove the cells from the plate. Next, astrocytes were rinsed with TBS then blocked in TBS with 1% goat serum on ice for 20 min. This was followed by incubation with the appropriate primary antibody including anti-GFAP (Dako #Z0334), anti-PDGF-Rα (eBiosciences #14140182), anti-NG2 (Millipore, Temecula, CA #AB5320), anti-VEGFR1 (R&D Systems #MAB471) and VEGFR-2 (R&D Systems #MAB443). The astrocytes were washed twice and then incubated with the appropriate FITC-conjugated secondary antibody (Jackson ImmunoResearch, West Grove, PA) diluted in TBS with 1% BSA and incubated on ice for 30 min. The resulting stained astrocytes were washed twice and resuspended in 0.5 ml of TBS with 1% BSA. The samples were analyzed with a FACScan caliber flow cytometer (Becton-Dickinson) and the resulting representative histograms are shown.

### VEGF analysis

The level of VEGF secreted was assessed using a Mouse VEGF Immunoassay kit (R&D Systems) as we previously described [18]. Astroglial cells (6 x 10^5^) were plated on a 60 mm tissue culture dish. Once the cells had reached ~90% confluence they were rinsed with serum-free DMEM and then grown in serum-free DMEM for 2 days. The conditioned medium was then clarified. The VEGF Immunoassay was performed in triplicate for the samples and normalized to the number of cells. A standard curve was generated to assess the sample VEGF levels. The assay was repeated twice with similar results.

### Apoptosis and proliferation

Astroglial cell apoptosis was determined using a Click-iT TUNEL AlexaFluor^TM^ 594 Imaging Assay (Invitrogen # C10246). Astroglial cells were grown on fibronectin coated (2 μg/ml) chamber slides in growth medium overnight. As an apoptotic challenge, cells were incubated with 100 nM Staurosporine (Invitrogen) in growth medium for 24 hours. The next day the chambers were washed twice with PBS, 4% paraformaldehyde was added for 20 min and the chambers were then again washed twice. Cell permeablization was accomplished by adding 0.5% Triton X-100 in PBS (10 min). The Click-iT TUNEL Imaging Assay was used as directed by the supplier and the astrocytes that stained positive were counted with the use of a fluorescent microscope. The percent of apoptotic cells relative to the total number of cells per 10 high power fields (x400) were recorded.

To assess cell proliferation, astroglial cell numbers were counted every other day for 2 weeks. Initially, 2 x 10^4^ astroglial cells were plated on multiple gelatin-coated 60 mm dishes such that triplicate counts could be determined for each time point for the entire experiment. A hemocytometer was used to count cells every other day for 9 days. The cells were then fed on alternate days to counting.

### Western blot analysis

Astroglial cells were plated at 6 x 10^5^ cells per 60 mm gelatin-coated tissue culture dish. After they were to ~90% confluence the plates were rinsed with serum-free DMEM and growth medium that did not contain serum was added. Two days later the conditioned medium (CM) was harvest and clarified. In addition, the cells were lysed in 0.1 ml of lysis buffer (50 mM HEPES pH7.5, 100 mM NaCl, 0.1 mM EDTA, 1 mM CaCl_2,_ 1 mM MgCl_2_, 1% Triton X-100, 1% NP-40 and protease inhibitor cocktail (Roche Biochemical, Mannheim, Germany). Protein content was determined and the samples adjusted appropriately with 6x SDS-sample buffer then 25 μg of total protein was analyzed using SDS-PAGE (4–20% Tris-glycine gels, Invitrogen). Proteins were transferred to a nitrocellulose membrane and the membranes blocked at room temperature (1 hour; 0.05% Tween-20 and 5% skim milk in TBS). The following antibodies were added at 1:1000 dilutions recommended by the supplier after blocking: anti-TSP1 monclonal antibody (MS-421P clone A6.1; NeoMarker, Fremont, CA), anti-osteopontin (AF808; R&D Systems), anti-SPARC (AF942; R&D Systems) and anti-β-actin (MAB-515739; Thermofisher), anti-phospho JNK (Thr221;#AF-1205; R&D Systems), anti-JNK (AF-1387; R&D Systems), anti-phospho Akt (Ser473;#9271; Cell Signaling Technology), anti-Akt (9272; Cell Signaling Technology), anti-phospho Erk 1/2 (Thr202;#9106; Cell Signaling Technology), anti-Erk (9102; Cell Signaling Technology), anti-phospho P38 (Thr180;#9211; Cell Signaling Technology), anti-P38 (9212; Cell Signaling Technology), anti-phospho Src (Tyr416;#2101; Cell Signaling Technology), anti-Src (2123p; Cell Signaling Technology), anti-phospho NFkB (Ser536;#3033; Cell Signaling Technology), anti-NFkB (8242; Cell Signaling Technology) and anti-β-tubulin (ab6046; Abcam). This was followed by washing, incubation with appropriate Peroxidase AffiniPure secondary antibodies (1:5000; Jackson ImmunoResearch) and development using Amersham ECL (GE, Pittsburgh, PA). For the densitometry, protein quantification was normalized to β-tubulin or β-actin (see figure for details) and then compared to wild-type control.

### Wound and transwell assay

For the wound assay, Culture-Insert 2 Well (cat #: 80206, Ibidi) was used to create a 500μm gap. Briefly, 3x10^5^ cells/ml suspension was prepared and 100ul was placed into each well. After appropriate cell attachment (24hr), the Culture-Insert-2 Well was gently remove using sterile tweezers. Cell monolayers were rinsed with DMEM containing 10% FBS twice and fed with growth medium containing 1 μM 5-fluorouracil (F6627, Sigma) to exclude potential contribution of cell proliferation to wound closure. Wound closure was monitored and photographed at 0, 24, and 48 h using a phase microscope in digital format. For quantitative assessment, the distances migrated as percent of total distance were determined.

As we previously described, fibronectin coated (2μg/ml in PBS) transwell filters (Corning #3422; 8 μm polycarbonate) were obtained following an overnight incubation at 4°C [18]. The transwell filters were then rinsed with PBS, blocked with 2% BSA at room temperature for 1 hour and then rinsed with PBS. Serum-free DMEM (500 μl) was added to the well bottom with 100 μl of astrocytes in serum-free DMEM (1 x 10^5^) added to the top. Four hours later the cells with medium was aspirated from the top and the remaining cells wiped off the upper chamber with a cotton swab. Next, astroglial cells that had migrated through the membrane were hematoxylin-eosin stained and mounted. For each condition, ten-high power fields were counted (x200) and the mean and standard deviation determined with samples done in triplicate.

### Cell adhesion

Adhesion of astroglial cells to collagen I, collagen IV, fibronectin and vitronectin (BD Bioscience) was accomplished by varying the concentration of the extracellular matrix proteins, by dilution in TBS with Ca^2+^Mg^2+^ (2 mM each), onto a 96-well plate (50 μl; Nunc MaxiSorp plates, Fisher Scientific) overnight at 4^0^ C. The plates were the rinsed (4x) with TBS with Ca^2+^Mg^2+^, blocked in 1% BSA in TBS with Ca^2+^Mg^2+^ (200 μL) for 1 hour and rinsed with TBS with Ca^2+^Mg^2+^. The astroglial cells were removed with 1.5 mL of dissociation solution, washed 1x with TBS, resuspended in HEPES-buffered saline (5 x 10^5^ cells/mL; 25 mM HEPES, pH7.6, 150m M NaCl, 4 mg/ml BSA) and 50 μL of cell suspension was added to each well which had 50 μL of TBS with Ca^2+^Mg^2+^. Samples were in triplicate. The cells were allowed to adhere at 37°C in a humid incubator for 90 minutes. To remove nonadherent cells, the plates were gently washed (4x) with 200 μL of TBS with Ca^2+^Mg^2+^ until no cells remained in the BSA only coated wells. To quantify the number of adherent cells, the levels of intracellular acid phosphatase was assessed by lysing cells in 100 μL of lysis buffer (50 mM sodium acetate pH 5.0, 1% Triton-X-100, 4 mg/mL p-nitrophenyl phosphate) and incubating overnight at 4°C. To neutralize the reaction 50 μL of 1M NaOH was added and the absorbance read on a plate reader (Thermomax, Molecular Devices) at 405 nm.

### Astroglial cell morphogenesis in Matrigel

Matrigel (0.5 mL; Corning #354234) was applied to the bottom of a 35 mm tissue culture dish to harden for 30 min at 37°C. Trypsin-EDTA was used to remove the cells. The cells were then washed with growth medium 1x and then resuspended at 2 x 10^5^ cells/mL in growth medium not containing FBS. Then 2 x 10^5^ cells in 2 mL was added to the Matrigel-coated plates and incubation continued at 33^0^ C for 18 hours. To quantitatively assess the data, we determined the mean numbers of branches under 5 high-power fields (x100) using the following software for analysis http://image.bio.methods.free.fr/ImageJ/?Angiogenesis-Analyzer-for-ImageJ&artpage=3-6#outil_sommaire_3. Note increasing the incubation time did not enhance branching morphogenesis.

### RNA purification and real time qPCR analysis

Total RNA from retinal astrocytes was extracted using mirVana PARIS kit (Invitrogen). Sprint RT Complete-Double PrePrimed kit (Clontech, Mountain View, CA) was used for cDNA synthesis with total RNA (1 μg) extracted from retinal astroglial cells using a mirVana PARIS kit (Invitrogen). For a template, cDNA (1 μl each diluted 1∶10) was used in qPCR assays which were performed in triplicate of three biological replicates on a Mastercycler Realplex (Eppendorf) with a SYBR-Green qPCR Premix (Clontech). Amplification parameters were as follows: 95°C for 2 min; 40 cycles of amplification (95°C for 15 sec, 60°C for 40 sec); dissociation curve step (95°C for 15 sec, 60°C for 15 sec, 95°C for 15 sec). Primer sequences used; Bmp7 5’-GG GCTGGTTGGTGTTTGA-3’ (forward) and Bmp7 5’-GATGCTCTGCCCATCCAG-3’ (reverse); MCP-1 5’-GTCTGTGCTGACCCCAAGAAG-3’ (forward) and MCP-1 5’-TGG TTCCGATCCAGGTTTTTA-3’ (reverse IL6 5’- CAACCAC GGCCTTCCCTACT-3’ (forward) and IL-6 5’-TTGGGAGTGGTATC CTCTGTGA-3’ (reverse) and RpL13a were 5′-TCTCAAGGTTGTTCG GCTGAA-3′ (forward) and Rpl13a 5′-CCAGAC GCCCCAGGTA-3′ (reverse). We generated standard curves from known quantities of each target gene with linearized plasmid DNA. We used ten times dilution series for each known target, which we amplified using SYBR-Green qPCR. Then the linear regression line for DNA (ng) was determined from relative fluorescent units (RFU) at a threshold fluorescence value (Ct). We were able to quantify gene targets from cell extracts by comparing the RFU at the Ct to the standard curve, normalized by the simultaneous amplification of RpL13a, a housekeeping gene.

### Statistical analysis

To evaluate statistical differences between groups we utilized ANOVA with Tukey’s Multiple Comparison Test and show the mean with standard error of mean (SEM). To evaluate statistical differences between wild-type and Bim -/- astroglial cells and confirm data pairs from groups a student’s unpaired *t-*test (2-tailed) was utilized. qPCR data shown here were analyzed with a student’s unpaired *t*-test. Data are presented as mean ± SEM. We considered P values ≤ 0.05 significant.

## Results

### Bim -/- retinal astroglial cells produced increased levels of VEGF

**Fig 1:**
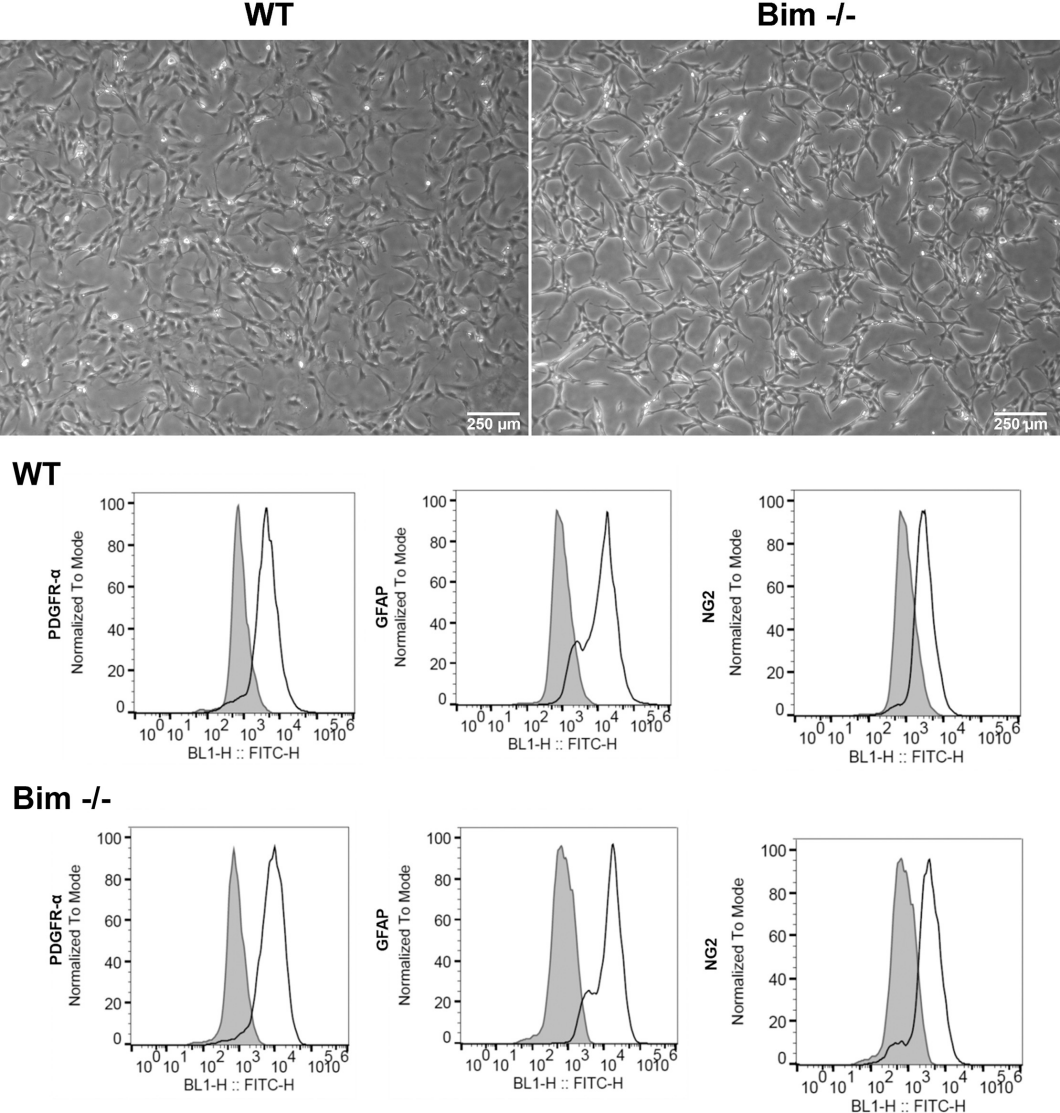
Wild-type and Bim -/- retinal astroglial cells. In Panel A, retinal astroglial cells from wild-type and Bim -/- mice were cultured on gelatin coated plates and photographed with a phase microscope in digital format. PDGFR-α, GFAP and NG2 expression was assessed by FACScan analysis. In Panel A, Scale bar = 250 μm. Please note minimal differences in morphology between wild-type and Bim -/- astroglial cells. The shaded histogram denotes staining in the presence of control IgG.

Retinal astrocytes lay a scaffold for endothelial cells and pericytes to go along to form the nascent retinal vascular network. Here we addressed the influence Bim expression has on basic astroglial cell properties essential for their unique function. Isolated retinal astroglial cells from wild-type (WT) and Bim -/- mice displayed minimal differences in morphology (Fig 1). To ensure that these cells maintained expression of cell specific markers we assessed expression of platelet derived growth factor-receptor α (PDGFR-α), glial fibrillary acidic protein (GFAP) and neuroglia proteoglycan 2 (NG2) by FACScan analysis. Astrocyte markers PDGFR-α, NG2 and GFAP were similarly expressed by both wild-type and Bim -/- astroglial cells (Fig 1).

**Fig 2:**
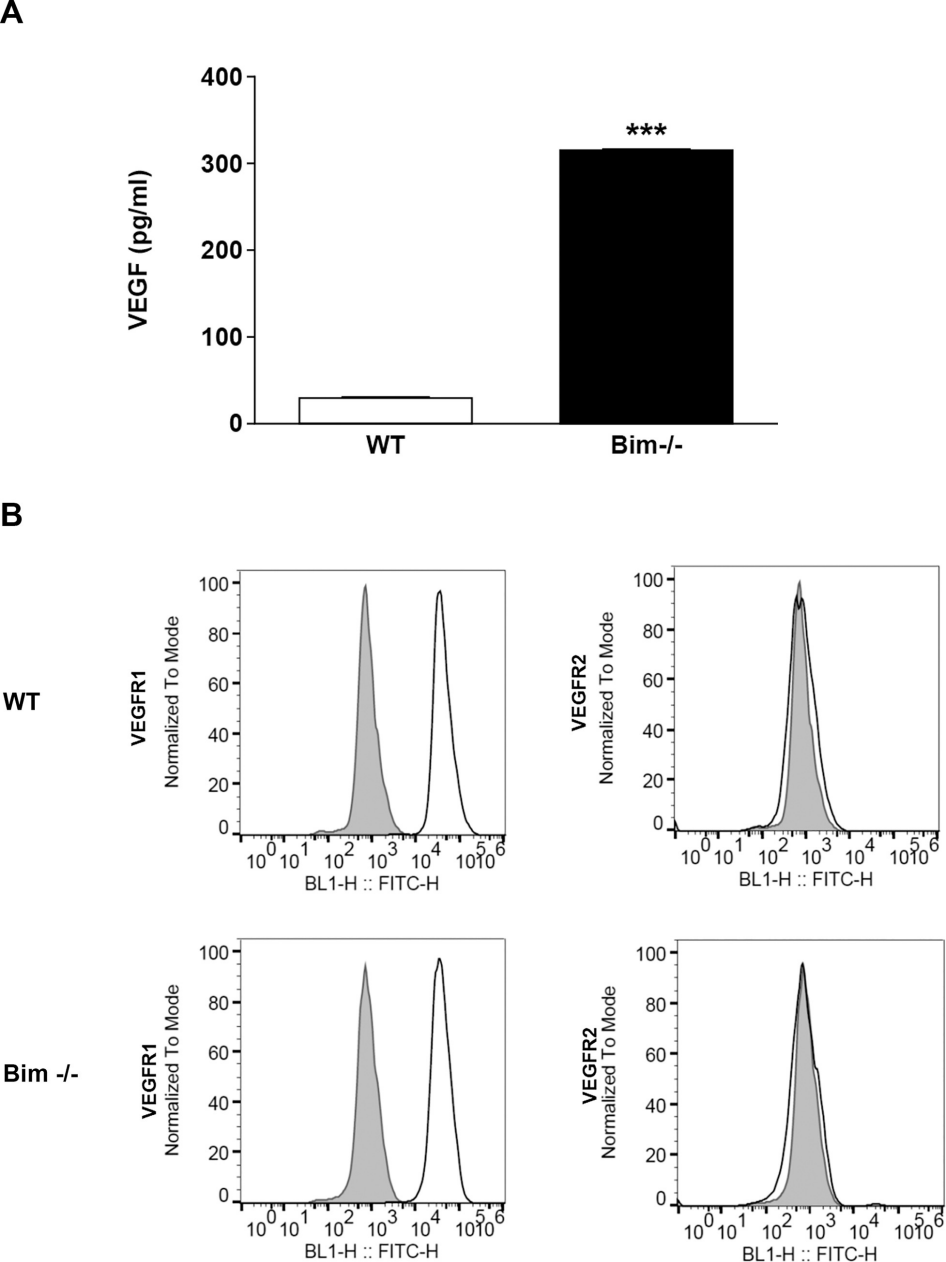
Increased VEGF expression in the absence of Bim. In Panel A, VEGF levels of wild-type and Bim -/- astroglial cells were determined utilizing an ELISA as described in the Methods section. In Panel B, FACScan analysis was employed to assess VEGFR1 and VEGFR2 expression in wild-type and Bim -/- astroglial cells. The shaded histogram denotes staining in the presence of control IgG. Please note increased VEGF expression in the absence of Bim. No VEGFR2 expression was noted in retinal astroglial cells. (P***< 0.001; n = 6; student’s unpaired *t-*test (2-tailed) was utilized).

VEGF expression plays a central role during developmental and pathological angiogenesis as well as during inflammation. In astrocytes, VEGF influences growth and differentiation promoting stabilization of the retinal vasculature potentially through modulation of proliferation and migration [25]. Here, we observed that lack of Bim expression in retinal astroglial cells resulted in approximately a 10-fold increase in VEGF expression in the absence of Bim (Fig 2A). VEGFR1, but not VEGFR2 expression, was noted in both wild-type and Bim -/- retinal astroglial cells (Fig 2B). Thus, increased VEGF expression in the absence of Bim did not correlate with VEGFR1 expression.

### Bim -/- astroglial cells proliferate at a faster rate

**Fig 3:**
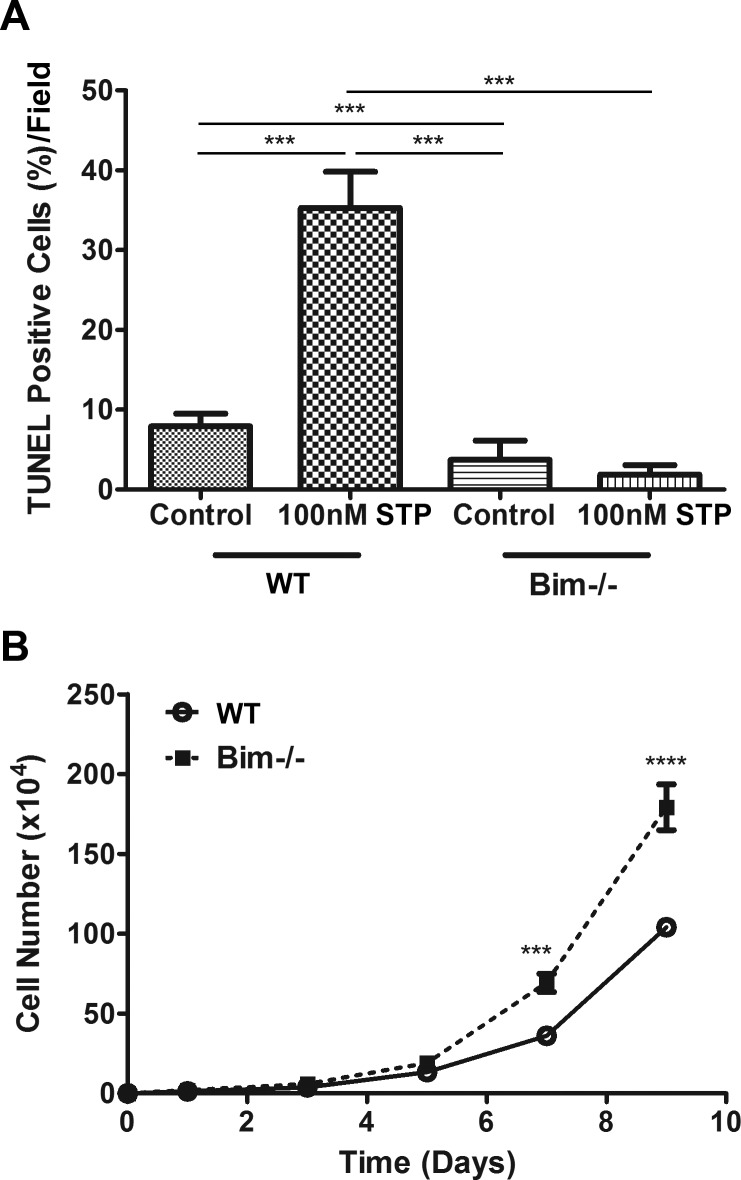
Modulation of apoptosis and proliferation. In Panel A, wild-type and Bim -/- astroglial cells were incubated with vehicle or 100 nM staurosporine (STP) for 24 h. Apoptosis was determined using a Click-iT TUNEL imaging assay and the percentage of TUNEL positive cells was determined in 5 high power field (x100). In Panel B, the growth rate of wild-type (ο) and Bim -/- (■) astrocytes was monitored over a 9 day period. These experiments were repeated three times with two different isolations of astroglial cells with similar results. (P***<0.001, P****<0.0001; n = 6; In Panel A, an ANOVA with Tukey’s Multiple Comparison Test was used and in Panel B a student’s unpaired *t-*test (2-tailed) was utilized.).

Since Bim expression is known to influence apoptosis, we next examined the basal and challenged (100 nM staurosporine; STP) level of apoptosis in wild-type and Bim -/- retinal astroglial cells. The basal level of apoptosis in wild-type astroglial cells was significantly higher than in Bim -/- cells. Upon challenge with 100 nm staurosporine, wild-type astroglial cells demonstrated a significant increase in the numbers of TUNEL positive cells. In contrast Bim -/- astroglial cells did not undergo increased apoptosis when challenged (Fig 3A). Next, we assessed proliferation by counting the number of retinal astroglial cells every other day for 9 days (Fig 3B). We noted a significant increase in cell numbers of Bim -/- astroglial cells compared to their wild-type counterpart starting at 7 days. Thus, Bim -/- retinal astroglial cells were resistant to apoptosis when challenged and demonstrated increased proliferation compared to their wild-type counterpart.

### Bim -/- astroglial cells display aberrant extracellular matrix protein expression

**Fig 4:**
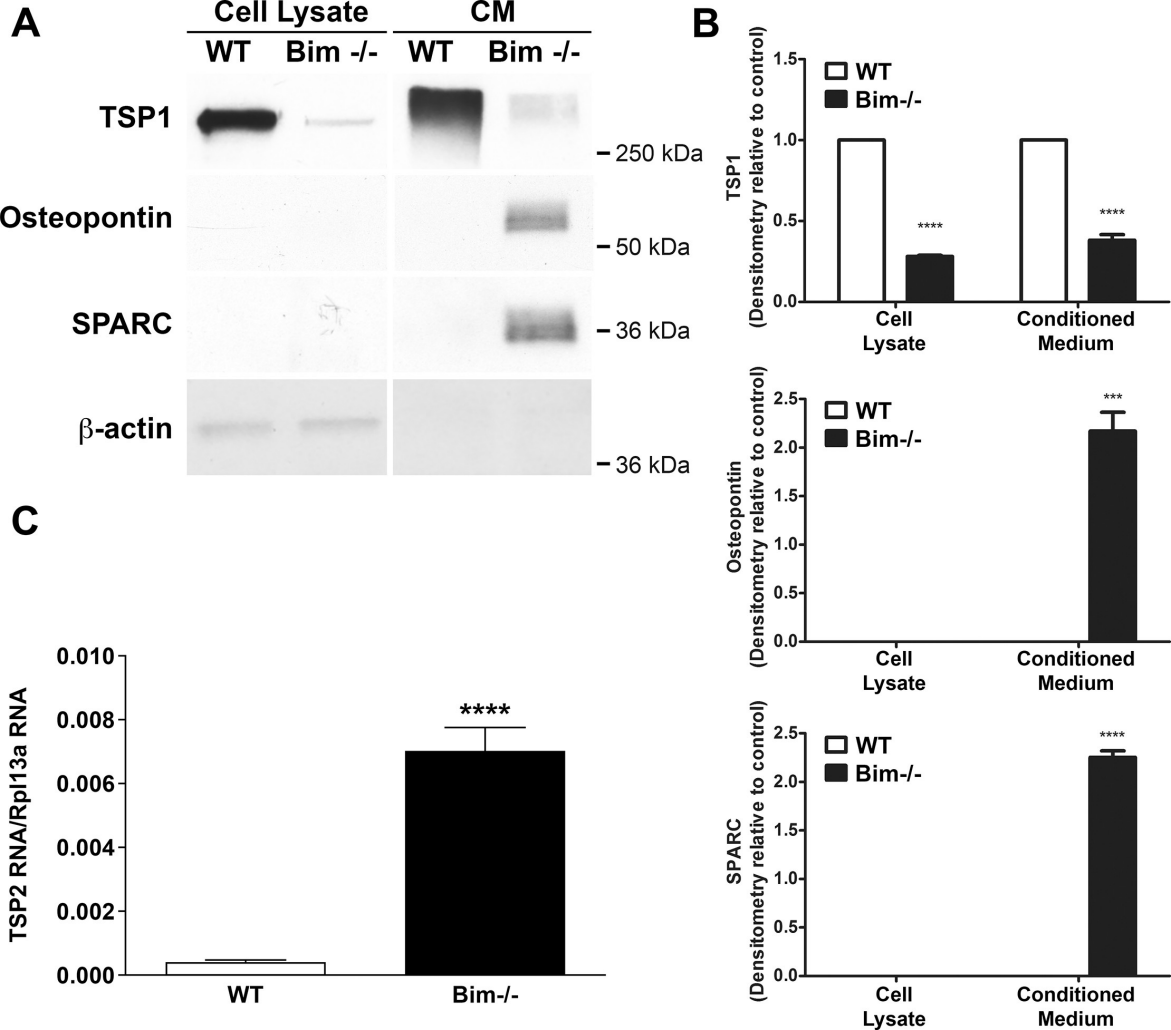
Bim -/- astroglial cells demonstrate increased secreted osteopontin and SPARC but decreased TSP1. Conditioned medium and cell lysates were collected at 48 hours for analysis of extracellular matrix proteins by Western blot analysis as described in Materials and Methods (Panel A and B). Panel A: The level of extracellular matrix proteins, secreted and cell associated, were assessed in wild-type and Bim -/- astroglial cells. Panel B: Quantification of band intensity for TSP1, osteopontin and SPARC. β-actin expression was assessed as a loading control. Panel C: qPCR analysis for TSP2. These experiments were repeated three times with two different isolations of astroglial cells with similar results. (P***<0.001, P****<0.0001; n = 6; In Panel B and C a student’s unpaired *t-*test (2-tailed) was utilized.).

The extracellular matrix composition influences vascular cell behavior and function, which can then directly affect angiogenesis. Next, expression of extracellular matrix proteins thrombospondin-1 (TSP1), osteopontin and SPARC was assessed by Western blot analysis of conditioned medium (secreted protein) and cell lysates (cell associated protein) (Fig 4A) from retinal astroglial cells. Osteopontin and SPARC were detected in the medium from Bim -/- astroglial cells but not wild-type cells. TSP1 demonstrated substantial expression both secreted (conditioned medium) and cell associated (cell lysate) from wild-type astroglial cells. In contrast, Bim -/- astroglial cells demonstrated modest TSP1 expression (Fig 4). The Western blots were quantified in Fig 4. Since TSP1 expression in astroglial cells is important for normal synaptogenesis of retinal ganglion cells, we next examined whether TSP2 expression in Bim -/- astroglial cells could compensate for the diminished TSP1 levels. Fig 4C shows increased TSP2 expression in the absence of Bim compared to levels in wild-type astroglial cells. Thus, lack of Bim expression changing the extracellular matrix milieu is consistent with a pro-inflammatory phenotype.

### Increased MCP-1 in the absence of Bim

**Fig 5:**
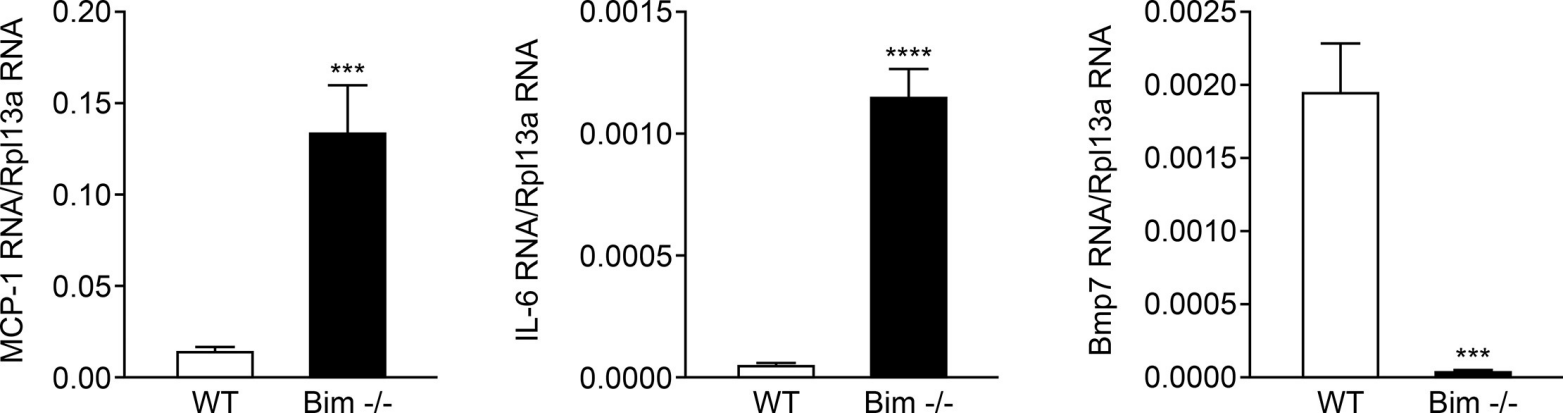
Increased MCP-1 by qPCR in Bim -/- astroglial cells. The expression levels of various inflammatory factors was assessed by qPCR analysis including MCP-1, IL-6 and Bmp7. These experiments were repeated three times with two different isolations of astroglial cells with similar results. Please note a significant increase in the levels of MCP-1 and IL6 (***P <0.001 ****P <0.0001; n = 6; Student’s unpaired *t*-test (2-tailed) was utilized) and decrease in Bmp7 levels in Bim -/- retinal astroglial cells (***P< 0.001; n = 6). RpL13A, 60S ribosomal protein L13a.

Increased osteopontin, SPARC and VEGF expression in Bim -/- astroglial cells may reflect altered expression of inflammatory mediators. To address this question we utilized qPCR to assess inflammatory mediator expression in retinal astroglial cells. We observed increased expression of monocyte chemoattractant protein-1 (MCP-1) and Interleukin 6 (IL6) but decreased bone morphogenic protein 7 (Bmp7) expression in Bim -/- retinal astroglial cells compared to their wild-type counterpart (Fig 5). No significant difference in expression of IL1β, INFγ, TNFα and iNOS was noted between wild-type and Bim -/- astroglial cells (not shown). Thus, lack of Bim expression correlated with altered MCP-1, IL6 and BMP7 expression.

### Bim -/- astroglial cells are less migratory

**Fig 6:**
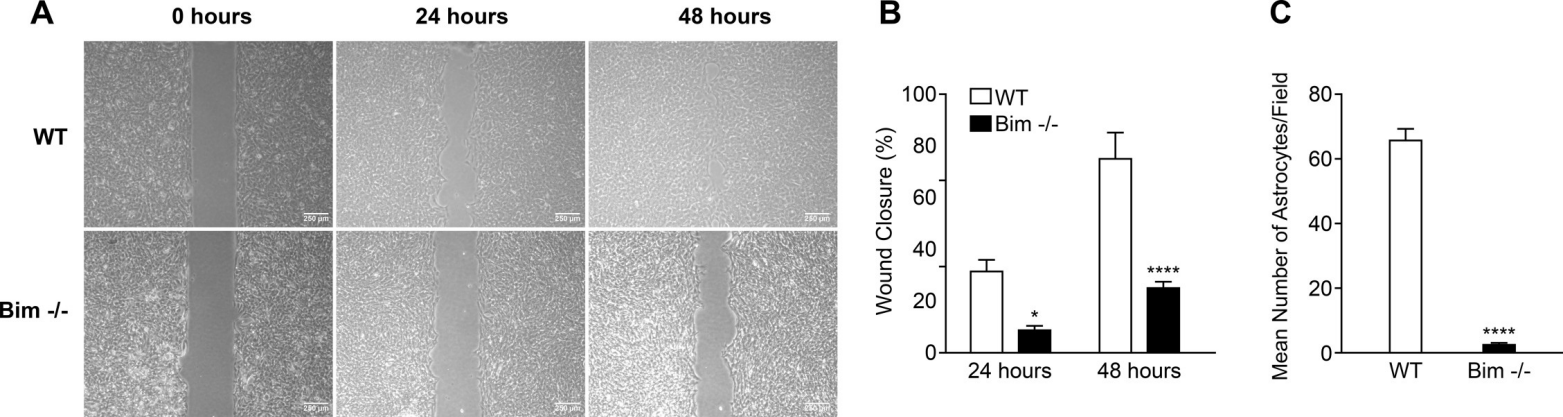
Bim -/- retinal astroglial cells demonstrate decreased migration. In Panel A, migration of wild-type and Bim -/- astroglial cells was determined using a scratch wound assay. Wound closure was monitored with a phase microscope in digital format at 0, 24 and 48 h. In Panel B, the percent closure of the wound was quantified at 24 and 48 h as compared to the zero hour time point. Note nearly complete wound closure with wild-type astroglial cells by 48 h while the Bim -/- astroglial cells lag behind. In Panel C, we utilized a transwell assay to monitor cell migration. Please note in the transwell assay Bim -/- astroglial cells also demonstrated less migration compared with wild-type astroglial cells. In Panel A, scale bar = 250 μm. These experiments were repeated three times with two different isolations of astroglial cells with similar results. (P*< 0.05, P****< 0.0001; n = 6; student’s unpaired *t-*test (2-tailed) was utilized).

Since extracellular matrix milieu impacts cell migration, critical during angiogenesis, we next assessed migration of wild-type and Bim -/- astroglial cells using a wound assay. Cells were grown to confluence around the Culture-Insert-2 Well after which it was removed revealing a uniform wound. The wounded monolayers were grown in the presence of 5-fluorouracil to prevent cell proliferation during the subsequent 48 hours. At 24 hours, wound closure was slightly but significantly delayed with Bim -/- astroglial cells compared to their wild-type counterpart. This delay in wound closure was more prominent by 48 hours (Fig 6A and 6B). Decreased migration of Bim -/- astroglial cells was also noted using a transwell migration assay (Fig 6C). Thus, lack of Bim expression in retinal astroglial cells correlated with decreased migration.

### Bim -/- astroglial cells demonstrate increased adhesion to fibronectin and vitronectin

**Fig 7:**
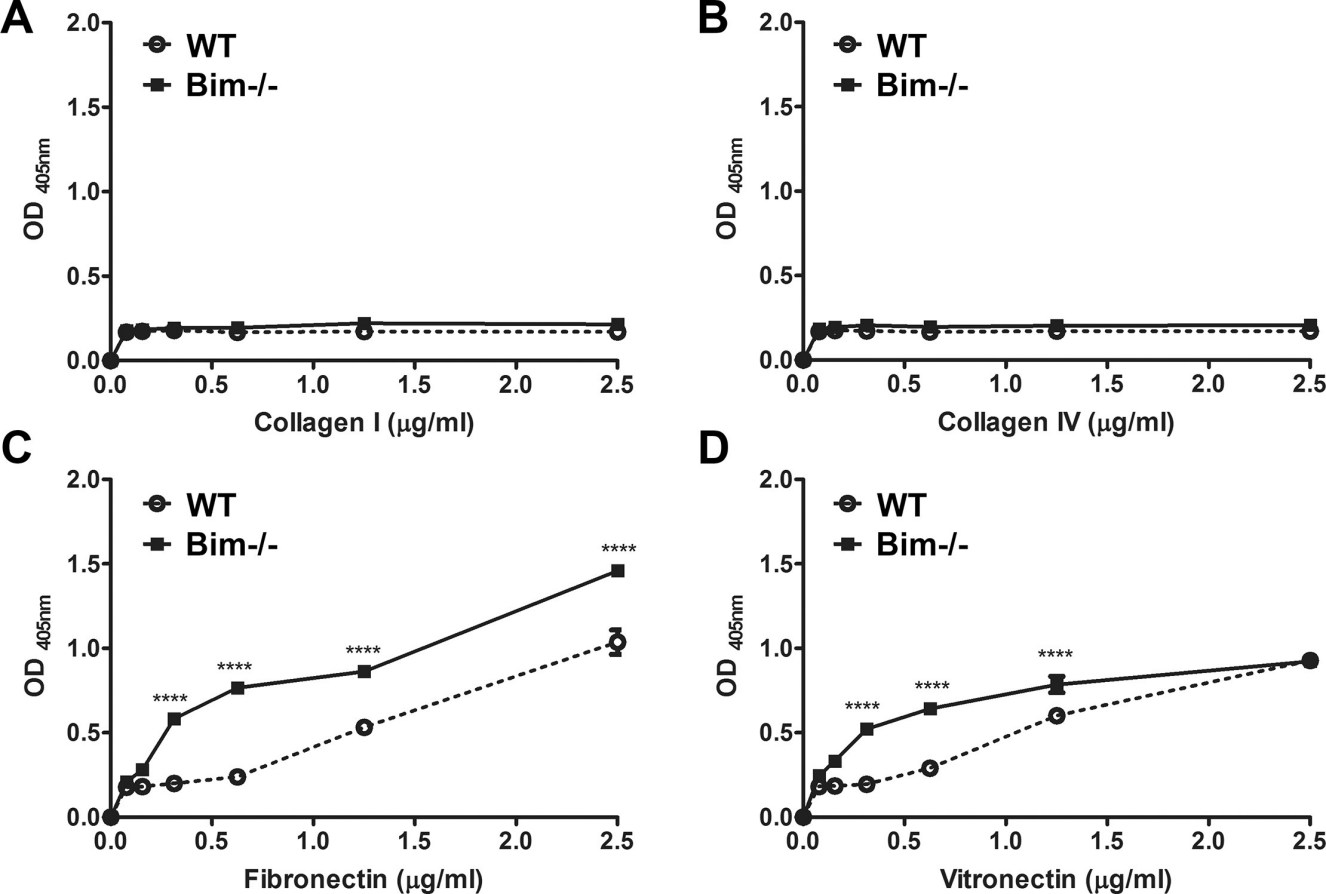
Bim -/- astroglial cells demonstrate more adhesion on fibronectin and vitronectin. Adhesion to varying concentrations of collagen type I (A), collagen type IV (B), fibronectin (C) and vitronectin (D) (0–2.5 μg/ml) was assessed with wild-type and Bim -/- astroglial cells. These experiments were repeated three times with two different isolations of astroglial cells with similar results. (wild-type (ο); Bim -/- (■); P****<0.0001; n = 6; student’s unpaired *t-*test (2-tailed) was utilized).

Altered cell migration can result from aberrant adhesive capacities to extracellular matrix substrates. Next, we examined the capacity of wild-type and Bim -/- astroglial cells to adhere to collagen I, collagen IV, fibronectin and vitronectin. Both wild-type and Bim -/- astroglial cells displayed minimal adhesion to collagen I or collagen IV. In contrast, considerable adhesion of Bim -/- astroglial cells to fibronectin and vitronectin was observed compared to wild-type astroglial cells (Fig 7). Thus, Bim deficiency increased astroglial cells adhesion to fibronectin or vitronectin.

### Bim expression facilitates morphogenesis of astroglial cells on Matrigel

**Fig 8:**
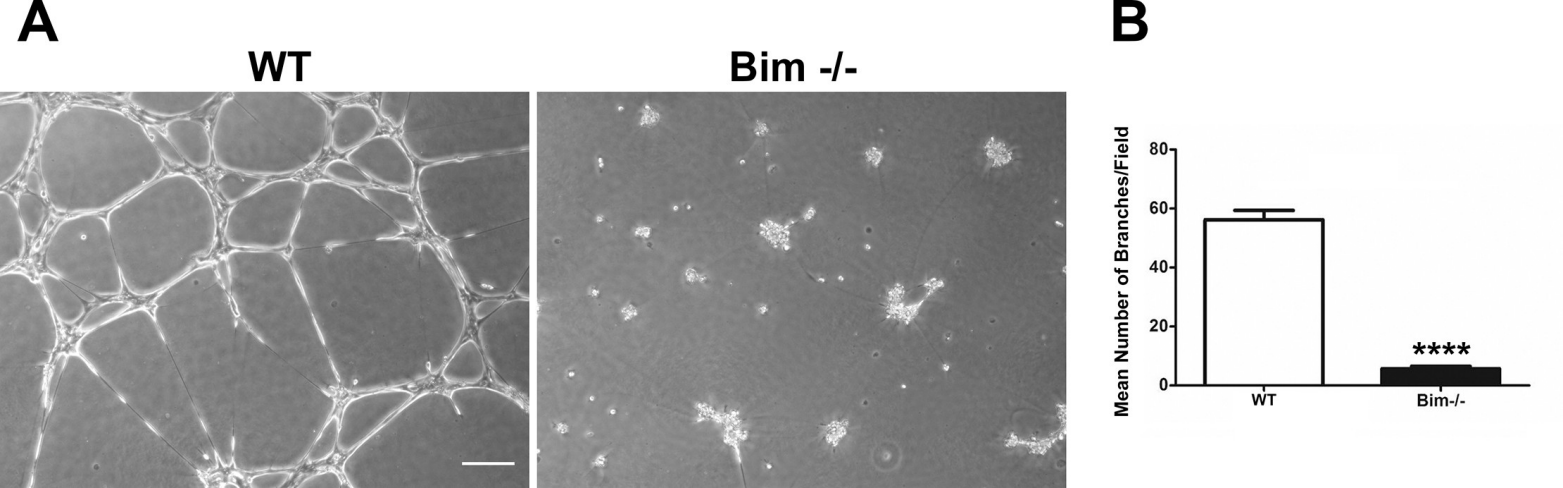
Diminished morphogenesis in Bim -/- astroglial cells. Wild-type and Bim -/- astroglial cells were plated on Matrigel and photographed in a digital format 18 hours later. The mean number of branches was determined and expressed as the mean number of branches ± standard deviation. These experiments were repeated three times with two different isolations of astroglial cells with similar results. Please note that the Bim -/- astroglial cells have only rudimentary morphogenesis in Matrigel. Scale bar = 250 μm. (P****<0.0001; n = 6; student’s unpaired *t-*test (2-tailed) was utilized).

Production of an astrocyte scaffold for retinal endothelial cells and pericytes to follow is essential during retinal vascularization and maintenance of a stable vasculature. To assess whether changes in extracellular matrix production, cell adhesion and migration impacts morphogenesis in the absence of Bim, we utilized a morphogenesis assay. Retinal astroglial cells were allowed to organize into a three dimensional-like network on Matrigel and the complexity assessed by quantifying the number of branches. Wild-type astroglial cells organized into a well-defined network as previously demonstrated [23]. In contrast, Bim -/- astroglial cells underwent rudimentary morphogenesis (Fig 8). Thus, lack of Bim expression in retinal astroglial cells diminished morphogenesis.

### Decreased phospho-Akt in the absence of Bim

**Fig 9:**
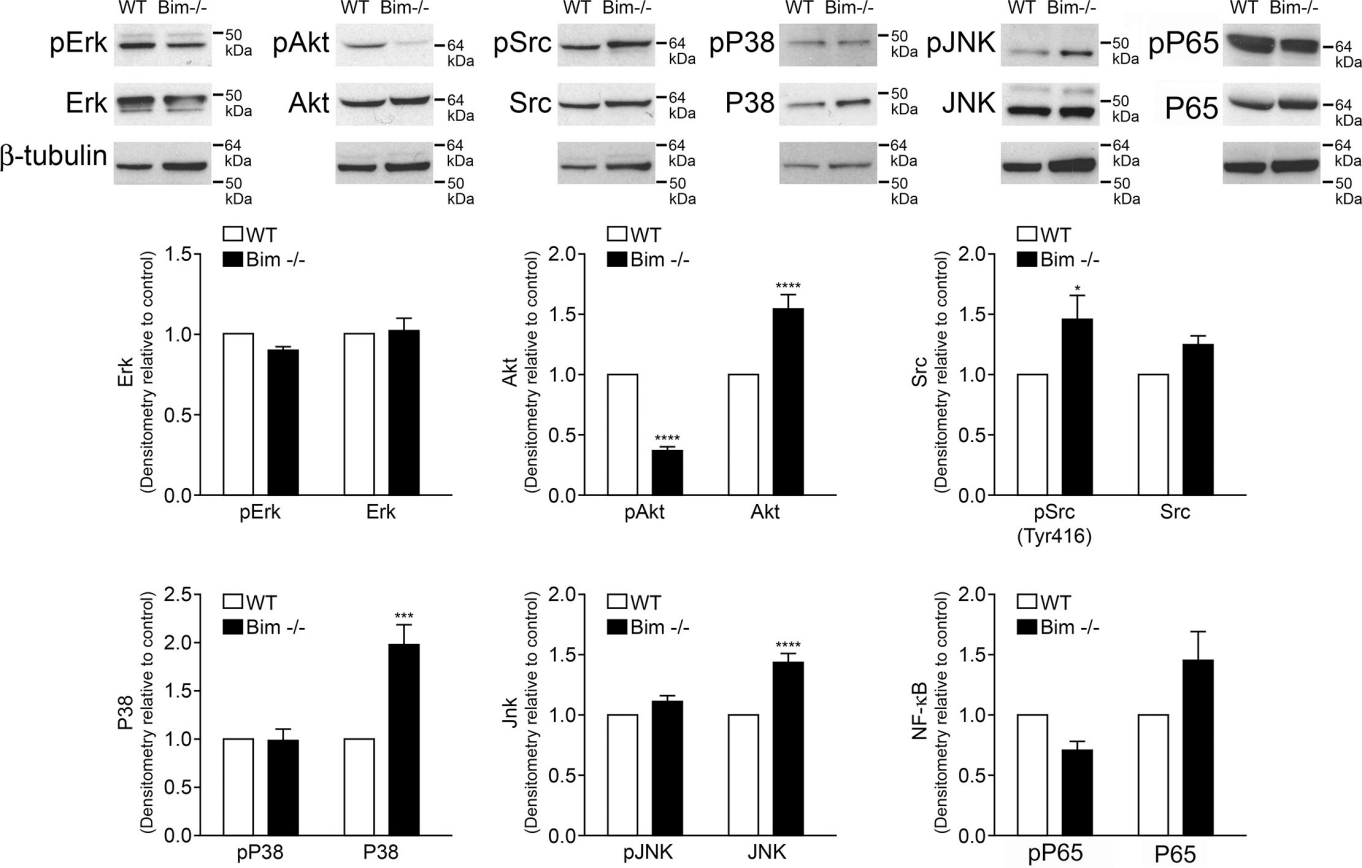
Decreased phospho-Akt expression in Bim -/- astroglial cells. Wild-type and Bim -/- astroglial cells were analyzed by Western blot analysis for expression of phospho-Erk1/2, total Erk1/2, phospho-Akt, total Akt, phospho-Src, total Src, phospho-P38, total P38, phospho-JNK, total JNK, phospho-P65 NF-κB, total P65 NF-κB and β-actin. These experiments were repeated three times with two different isolations of astroglial cells with similar results. A quantitative assessment of the data is also given. (*P < 0.05, ***P <0.001, ****P <0.0001; n = 6; student’s unpaired *t-*test (2-tailed) was utilized).

Our data demonstrate decreased apoptosis with challenge and increased proliferation, adhesion and expression of inflammatory mediators in Bim -/- astroglial cells compared to wild-type cells. Since mitogen activated protein kinases (MAPK), Akt, and Src signaling pathways influence these processes we assessed the impact lack of Bim expression had on these signaling pathways. Here we show decreased phospho-Akt expression in Bim -/- retinal astroglial cells compared to wild-type cells (Fig 9). We also observed increased Akt, P38, Jun N-terminal Kinase (JNK) and phospho-Src expression in Bim -/- astroglial cells. Thus, lack of Bim expression in retinal astroglial cells reduced the ratio of phosph-Akt to Akt with minimal impact on activation of MAPK pathways.

## Discussion

Retinal astroglial cells act in an intermediary-like capacity facilitating communication between neuronal cells and the retinal vasculature [1, 26]. The astrocytes of the retina emerge from the optic disc to lay down the fundamental framework that the nascent retinal vasculature will follow. If the neuronal cells are compromised, retinal astrocytes will not emerge from the optic disc and ultimately angiogenesis will not proceed [1, 25, 27]. The capacity of these astrocytes to form a cohesive durable network relies not only on their capacity to survive, but also to proliferate and migrate [28]. Retinal vascularization is attenuated if astrocytes are ablated demonstrating their essential role in angiogenesis [29]. Furthermore, lack of astrocyte VEGF expression prevents radial migration of endothelial cells during retinal vascular development [30]. Thus, angiogenesis driven vascularization of the retinal layers lays squarely with retinal astrocytes having optimal functional characteristics.

Here we assessed whether lack of Bim expression influences astroglial cell function. Bim and Bcl-2 are well known for their ability to modulate apoptosis in an opposing fashion, with Bim inducing apoptosis while Bcl-2 inhibits it [31–33]. In addition to the well-established role Bim plays in modulating apoptosis, our previous studies demonstrated that lack of Bim expression typically increased cell adhesion and VEGF expression [17–19]. We also have shown that Bim expression influences extracellular matrix expression, migration and proliferation in a cell type specific manner [17–19]. Our *in vivo* studies demonstrated that Bim -/- mice had increased retinal vascular density, precocious deep vascular plexus formation, decreased retinal vascular cell apoptosis and proliferation and attenuated hyaloid vessel regression [34]. These mice also did not undergo hyperoxia-mediated vessel obliteration and retinal neovascularization during oxygen-induced ischemic retinopathy. When Bim was only deleted in endothelial cells or pericytes we observed attenuated retinal postnatal vascular remodeling, perhaps due to decreased rate of apoptosis. Although hyaloid vessel regression was attenuated in mice lacking Bim expression in endothelial cells or pericytes, they responded similar to their control littermates to oxygen-induced ischemic retinopathy [16]. Thus, a better understanding of the implications to angiogenesis associated with these changes are emerging.

Cell migration has an integral and highly orchestrated role during normal tissue development and homeostasis, tissue wound repair and in pathologic conditions. Following tissue maturation, astrocytes are largely quiescent but revive their migratory capacity with injury and under pathologic conditions. In the retina, astrogliosis with resulting glial scar formation is the consequence of astrocyte activation [35, 36]. Here, we show that retinal astroglial cells lacking Bim expression have delayed migration. This is in contrast to what we had previously observed in Bim -/- retinal endothelial cells and pericytes which demonstrated increased migration [18]. VEGF expression can increase endothelial cell migration. Although Bim -/- astroglial cells demonstrated ~10-fold increase in VEGF expression (~300 pg/ml) compared to wild-type astroglial cells, they displayed decreased migration and no detectable VEGFR2 expression. Our previous studies in retinal endothelial cells and pericytes noted increases in VEGF expression in the absence of Bim in the 3-fold range, with the highest level noted in Bim -/- retinal pericytes (~120 pg/ml) corresponded with an increase in migration [18]. Thus, Bim -/- astroglial cells express higher VEGF levels than that observed in other retinal vascular cells, which did not correlate with changes in VEGFR2 expression. Thus, decreased astroglial migration in the presence of significantly increased VEGF expression should cause changes in astroglial cell function to ensue.

Osteopontin is a matricellular protein that plays multifaceted roles during development and in pathological states by influencing migration, adhesion and survival of many cell types including vascular and inflammatory cells. Here, we observed significantly increased osteopontin expression in Bim -/- astroglial cells corresponded to decreased migration perhaps due to these cells increased adhesive capacity. We also noted that Bim -/- astroglial cells were unable to undergo branching morphogenesis in Matrigel. We have observed a similar inability to undergo branching morphogenesis of endothelial cells that displayed decreased migration due to gene disruption [17, 37]. We also have previously shown disrupted branching morphogenesis of Bim -/- retinal pericytes co-cultured with wild-type retinal endothelial cells was attributed in part to excessive VEGF expression by utilizing a sFlt1 as a VEGF trap [18]. However, similar use of sFlt1 here did not improve branching morphogenesis of Bim -/- astroglial cells (not shown) indicating VEGF expression may not be sufficient to disrupt branching morphogenesis in these cells. This is consistent with the lack of VEGFR2 expression in astroglial cells.

The matricellular protein, TSP1, normally plays an important role during retinal neuronal synaptogenesis. The importance of TSP1 during synapse development is illustrated by studies showing addition of purified TSP1 increases ganglion cell synapse numbers [38] as well as localization of increased TSP1 expression to astrocytes near blood vessels following ischemia [39]. Here we showed a dramatic decrease in TSP1 expression in Bim -/- astroglial cells. TSP2 expression was upregulated in these cells perhaps as a compensatory change to stabilize neuronal synapses. We also observed that SPARC, another matricellular protein that negatively regulates synapses was upregulated. SPARC can bind VEGF, which is plentiful in Bim -/- astroglial cells. Thus, our studies suggest Bim expression in astroglial cells may aid neuronal synapse formation and function.

MCP-1 is a key chemokine that regulates migration and infiltration of different cell types [40]. MCP-1 released from various cells, exerts a potent pro-inflammatory effect on its target cells by binding to the CCR2 receptor [41]. Astroglia-derived MCP-1 induces migration of microglia leading to pathological microgliosis and inflammatory activation [42]. In addition, attenuation of MCP-1/CCL2 signaling in CCR2−/− mice increases astrocyte proliferation during brain injury reducing scar formation due to diminished extracellular matrix deposition [43]. IL-6 acts as both a pro- and an anti-inflammatory chemokine. During inflammation, IL-6 drives the switch from neutrophil to monocyte recruitment by enhancing monocyte-attracting chemokines such as MCP-1 [44–46]. Here we showed increased expression of MCP-1 and IL-6 in Bim-/- astroglial cells. This is consistent with studies showing lack of Bim expression affects inflammation [47–49]. Conversely, we observed decreased Bmp7 expression in Bim-/- astroglial cells. BMPs are growth factors that belong to the transforming growth factor beta (TGF-β) superfamily [50]. BMP7 triggers gliosis in both the Müller cells and astrocytes through microglia activation [35, 36]. BMP7 also antagonizes TGF-β mediated pro-inflammatory and pro-fibrotic activity [51]. We also demonstrated that loss of Bim expression in astroglial cells resulted in increased proliferation concomitant with a decreased rate of apoptosis, both under basal and challenged conditions. Thus, Bim expression not only squelches the inflammation process by clearing inflammatory cells, its expression also influences chemokine expression and thus has the potential to restrain chronic inflammation.

## Supporting information

S1 Raw Images (PDF)
