# Supplementary material for: Bim expression modulates the pro-inflammatory phenotype of retinal astroglial cells
Source: PLoS One. 2020 May 4;15(5):e0232779. doi: 10.1371/journal.pone.0232779 (PMC7197808; doi:10.1371/journal.pone.0232779)
Supplement: S1 Raw Images — (PDF) [file pone.0232779.s001.pdf]

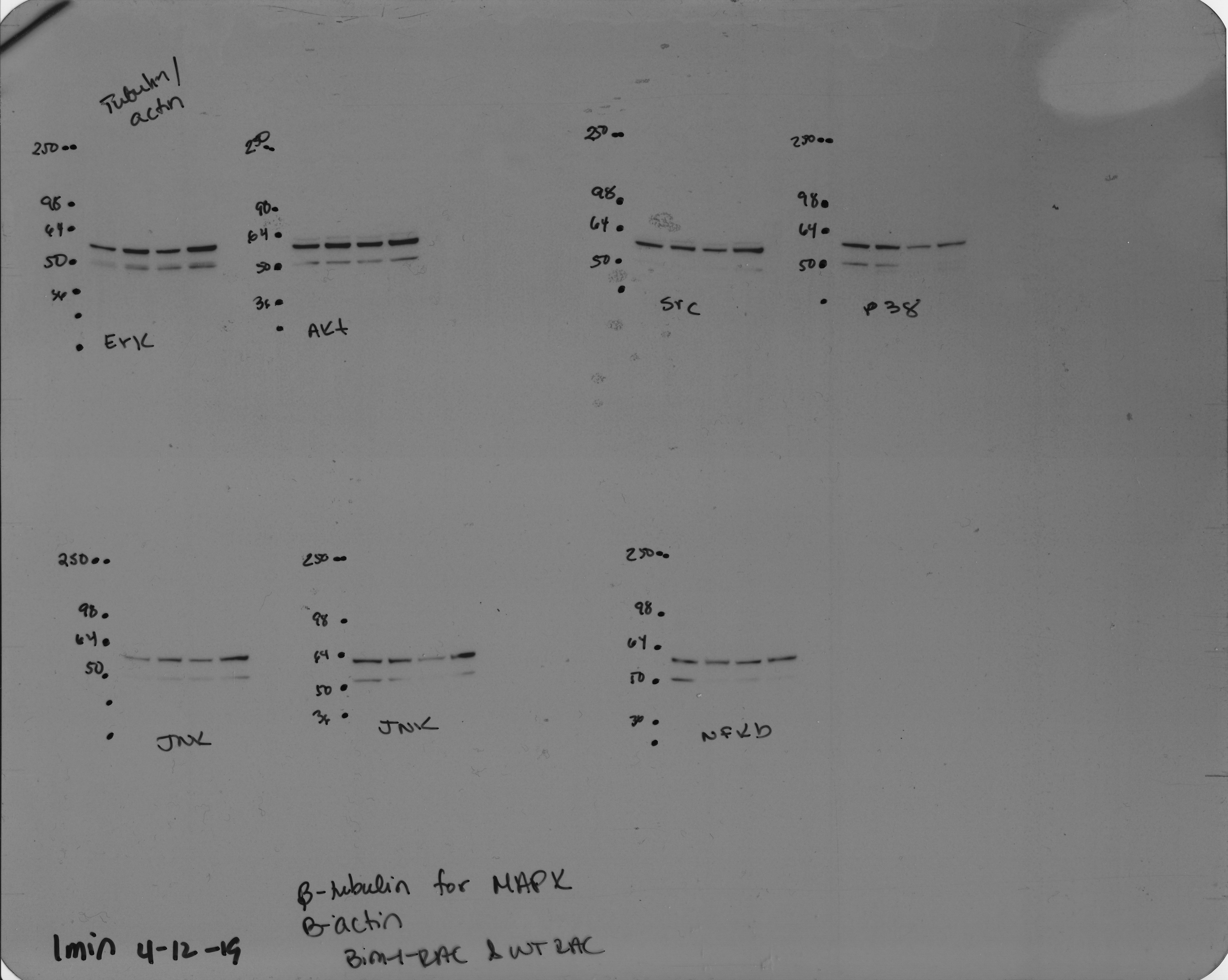

Tubulin/ actin
250
98
64
50
Erk
Akt
Src
p38
JNK
JNK
NFkb
β-tubulin for MAPK
B-actin
Bim1-RAC & WT RAC
1min 4-12-19

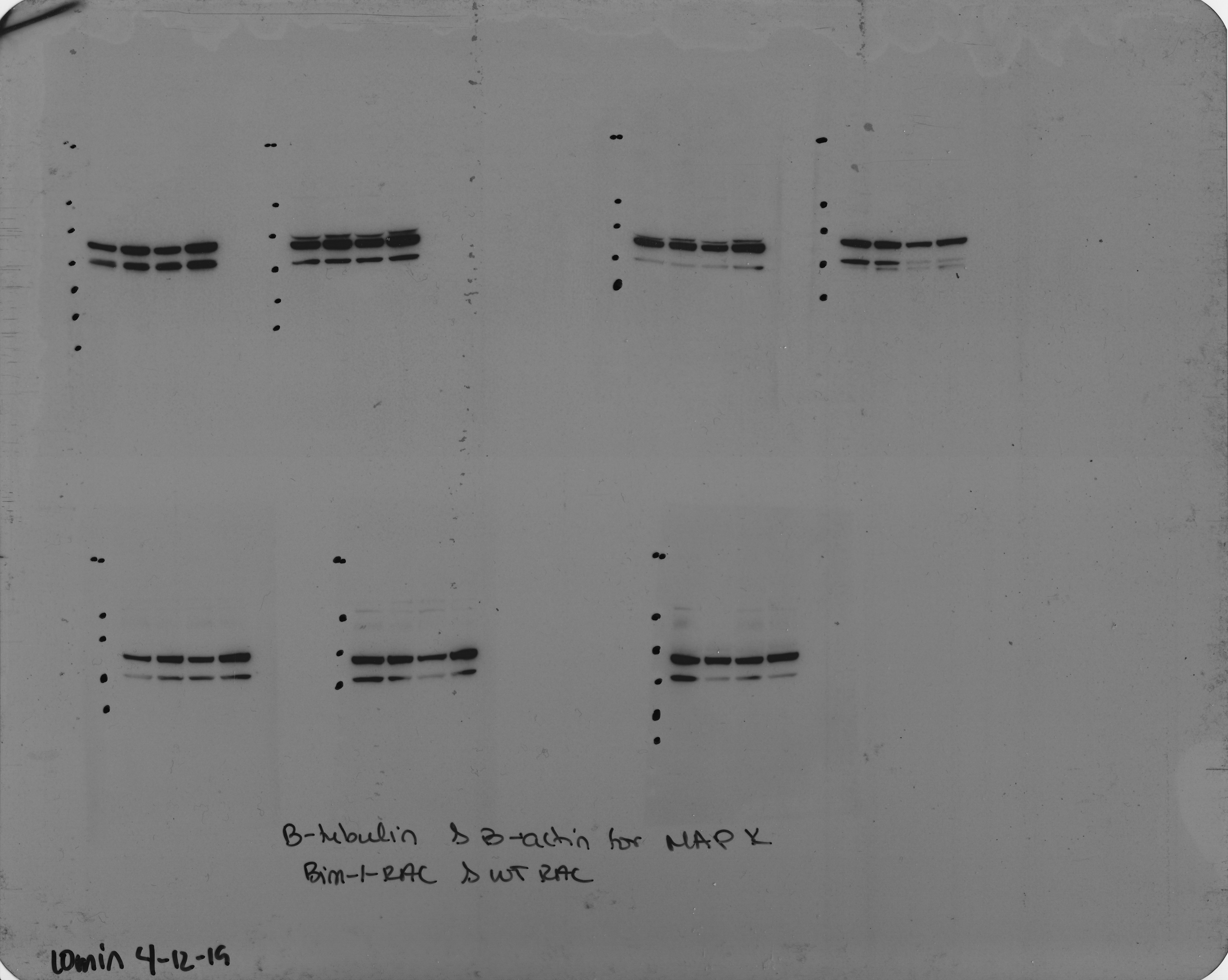
B-tubulin & B-actin for MAPK
Bim-1-RAC & WT RAC
10min 4-12-19

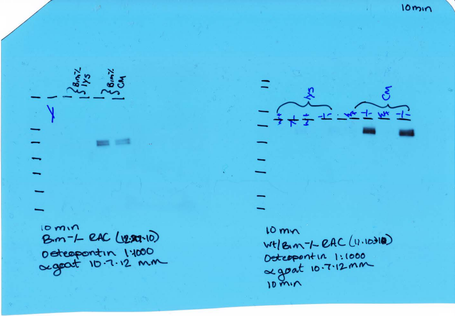
10min
Bim-/- lys
Bim-/- CM
lys
CM
wt
-/-
10 min
Bim-/- EAC ([illegible]-10)
Osteopontin 1:1000
α goat 10.7.12 mm
10 min
wt/Bim-/- EAC (11.10.[illegible])
Osteopontin 1:1000
α goat 10.7.12 mm
10 min

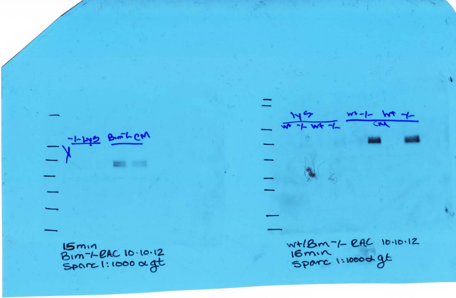
-/- lys
Bim-/- CM
15min
Bim-/- EAC 10.10.12
Sparc 1:1000 α gt
lys
wt -/- wt -/-
wt -/- wt -/-
CM
wt/Bim-/- EAC 10.10.12
16min
Sparc 1:1000 α gt

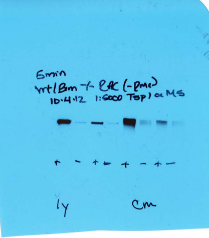
5min
wt/Bm -/- PAC (-pmc)
10.4.12 1:5000 Tsp1 α MS
+ – + – + – + –
ly
Cm

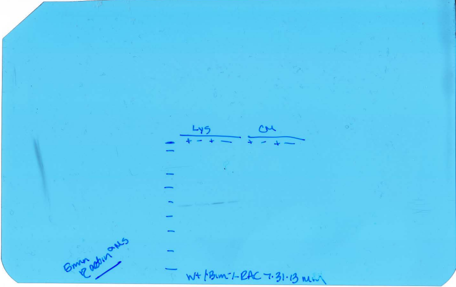
Lys
CM
+ - + -
+ - + -
5min
Reactions
Wt /Bim-/-RAC 7.31.13

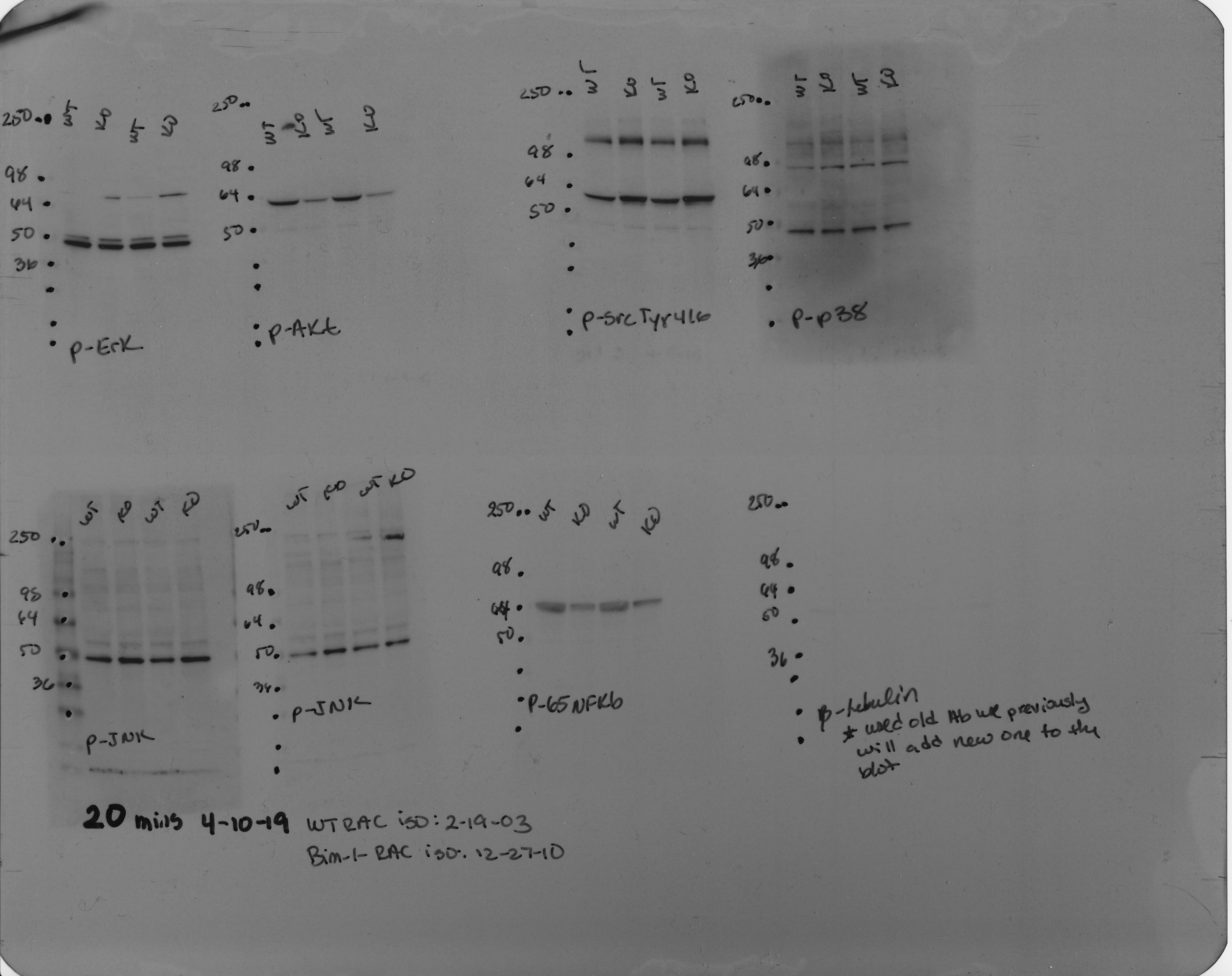
p-ErK
p-AKt
p-SrcTyr416
p-p38
p-JNK
p-JNK
p-65NFKb
β-tubulin
* used old Ab we previously
will add new one to the blot
20 mins 4-10-19 WT RAC iso: 2-19-03
Bim-1- RAC iso. 12-27-10

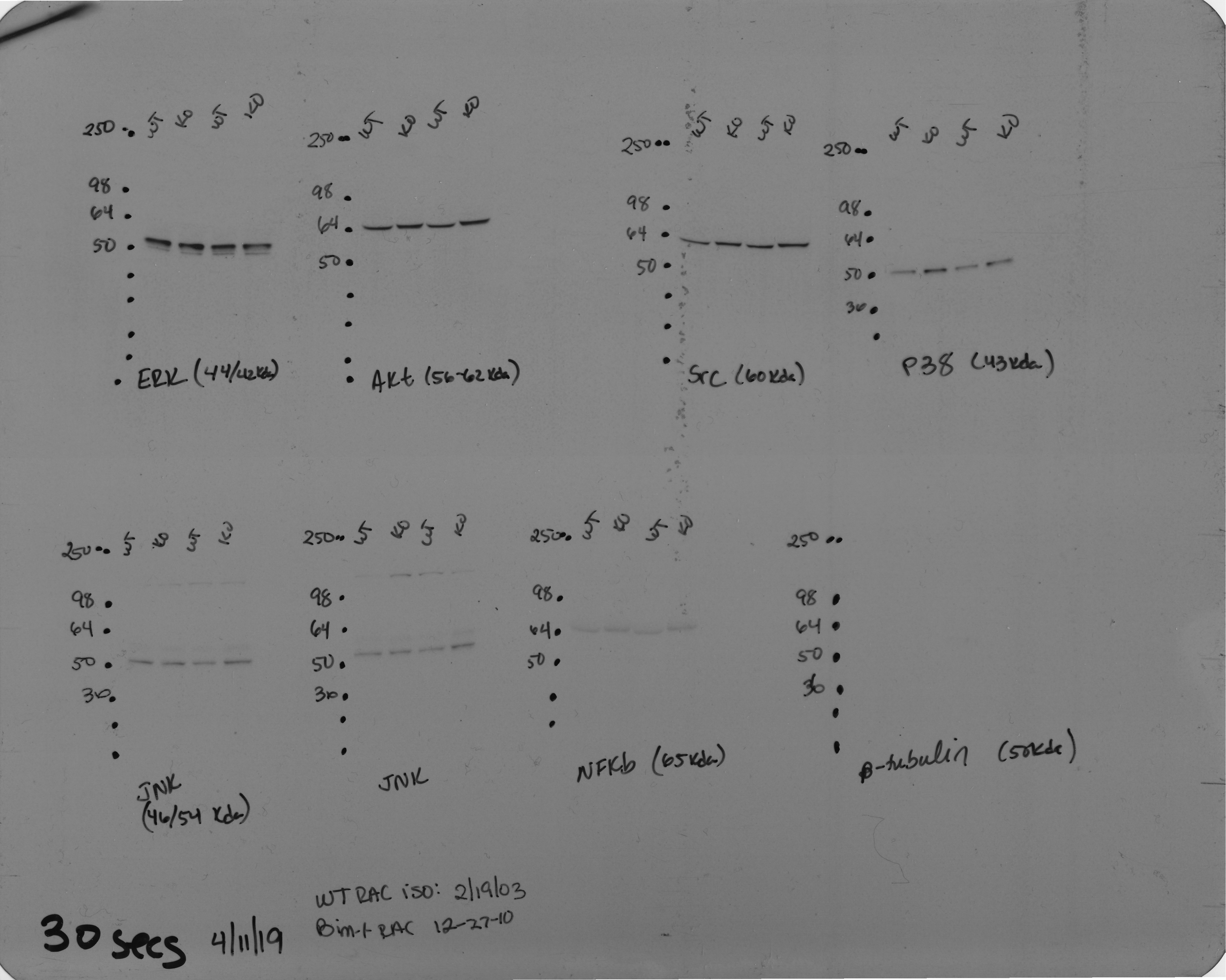

250
98
64
50
WT KO WT KO
ERK (44/42kDa)
AKt (56-62 kDa)
Src (60kDa)
P38 (43kDa)
30
JNK (46/54 kDa)
JNK
NFKb (65kDa)
β-tubulin (50kDa)
WT RAC iso: 2/19/03
Bim-/- RAC 12-27-10
30 secs 4/11/19

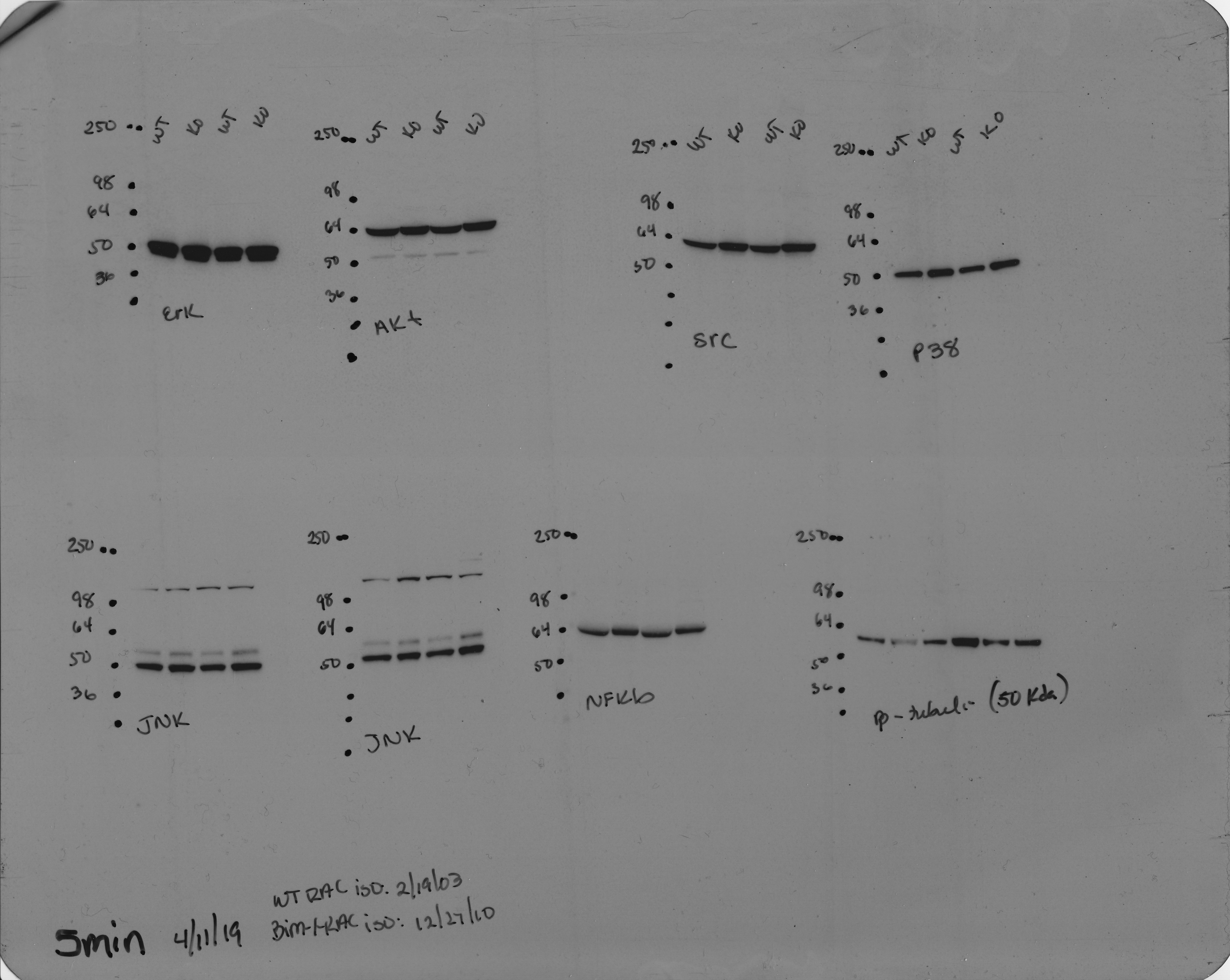
250
WT KO WT KO
98
64
50
36
Erk
AKt
Src
P38
JNK
JNK
NFKb
β-tubulin (50 Kda)
WT RAC iso. 2/19/03
Bim-/-RAC iso: 12/27/10
5min 4/11/19
